# ﻿Introducing multiple factor analysis (MFA) as a diagnostic taxonomic tool complementing principal component analysis (PCA)

**DOI:** 10.3897/zookeys.1248.159516

**Published:** 2025-08-04

**Authors:** L. Lee Grismer

**Affiliations:** 1 Herpetology Laboratory, Department of Biology, La Sierra University, 4500 Riverwalk Parkway, Riverside, California 92505, USA La Sierra University Riverside United States of America; 2 Department of Herpetology, San Diego Natural History Museum, PO Box 121390, San Diego, California, 92112, USA San Diego Natural History Museum San Diego United States of America

**Keywords:** Diagnosis, herpetology, meristic data, morphometric data, multivariate statistics, statistical defensibility, taxonomy

## Abstract

Multiple factor analysis (MFA) is introduced as a diagnostic tool for taxonomy and discussed using examples from the herpetological literature. Its methodology and output are compared and contrasted to the more often used principal component analysis (PCA). The most significant difference between MFA and PCA is that the former can more appropriately integrate numeric (meristic and/or morphometric) and categorical characters (e.g., big-small, blue-red, striped-banded, keeled-smooth, etc.) in the analysis, thus creating a nearly total-evidence morphological output. MFA emphasizes the diagnostic utility of categorical characters in a statistically defensible landscape as opposed to their often-anecdotal treatment or complete omission in species diagnoses, usually owing to their variability. PCA is most informative when only a single numeric data type (e.g., morphometric or meristic) is analyzed. Using PCA to analyze different data types separately and comparing the results, one can determine which data type and which of their variables (traits/characters) bear most heavily on the differentiation among the operational taxonomic units (OTUs [i.e., populations or species]) and, in some cases, their biological significance. If more than one data type is used in a PCA, the output may be biased by the data type with the largest amount of variation or statistical variance. Also discussed is the necessity of using a non-parametric permutation of analysis of variance (PERMANOVA)—or a similar analysis—as a robust, statistically defensible method for assessing the significance of OTU plot positions as opposed to subjective visual interpretations.

## ﻿Introduction

Even before the development of modern biology, morphological characters have been the evidential foundation upon which species were compared and diagnosed. Despite this longstanding convention, however, morphological diagnoses are rarely the result of rigorous statistical evaluation—quite notably so for herpetological taxonomy (see Chan and Grismer 2021). This stands in stark contrast to the advent of modern statistical theory and a plethora of analyses available for recovering statistically defensible significant differences between and among operational taxonomic units (OTUs [i.e., populations or species]). In this regard, the methodology for diagnosing and comparing species based on morphological data lags far behind the increasingly complex and ever-advancing methodologies used to delimit species using molecular datasets (e.g., Finger et al. 2021; Davis et al. 2023; Chambers et al. 2025)—and as such, has yet to progress beyond the techniques of some of the great 19^th^ century taxonomists such as George A. Boulenger and Edward D. Cope. But because journals continue to publish species descriptions that lack any sort of statistical diagnostic rigor (such as my own up through 2016), Chan and Grismer (2021: fig. 2) provided a simple 5-step univariate workflow to test for and visualize statistically significant diagnostic morphological differences between and among OTUs. This is augmented here with a demonstration of the broad utility of multivariate analyses as a diagnostic taxonomic tool. Of the many types of multivariate methodologies, principal component analysis (PCA) is the most commonly used in herpetological taxonomy. Therefore, it is compared and contrasted here with the more versatile and informative multiple factor analysis (MFA) whose first use in *any* taxonomic paper was quite recent (Grismer et al. 2021). Also discussed is the rationale for the necessity of a non-parametric permutation analysis of variance (PERMANOVA) as a robust statistical alternative to the more commonly used “subjective visualization” technique (i.e., “eyeballing it”) for assessing the significance of OTU clustering in multivariate space.

Overall, these analyses can augment a taxonomist’s ability to construct statistically defensible classifications based on both multivariate and standard univariate analyses (i.e., Student t-tests, Welch’s t-test, Mann-Whitney U test, various types of ANOVAs). This review is not intended to be an exhaustive overview of the multitude of currently available multivariate methods in this increasingly complex and growing landscape (see Paliy and Shankar 2016) or the mathematics involved in their analyses. Rather, it is to compare the utility and differences between PCA and MFA in order to emphasize the usefulness of multivariate analyses in taxonomic research and to provide background information that will help researchers in the selection of one over the other.

## ﻿Overview of multivariate analyses and their diagnostic utility

Multivariate ordination analyses summarize and visualize complex datasets by arranging data points (i.e., characters/traits/variables/metrics/morphometirs) along a few key axes (components or dimensions) that represent underlying gradients or patterns in the data. In general, multivariate analyses are equally exploratory as they are inferential. They can provide researchers with initial hypotheses and predictions as to how individual samples (specimens) may cluster together in morphospace (i.e., their position in a scatter plot) based on a set of input characters (usually morphology but genetic, physiological, ecological, and other data may also be used)—and more specific to taxonomy, how these OTU clusters may align with their phylogenetic delimitation. The most commonly used ordination procedure in herpetological taxonomy is PCA. Like other multivariate analyses, PCA reduces the dimensionality (i.e., number of variables/characters) of large complex datasets while graphically arranging the most signal-rich data along a few independent axes (i.e., components) that—as noted above—recover the majority of the underlying variation in the dataset. In other words, it provides the best signal-to-noise ratio (i.e., informative data-to-uninformative data ratio) for descriptive interpretations (Skalski et al. 2018) and potential diagnoses. MFA functions much the same way as other ordination analyses and when it was first introduced as a taxonomic tool for herpetology (Grismer et al. 2021), it was shown to be a more discriminating alternative to PCA (see below), largely owing to its ability to use a wider range of data types that contained additional information and discriminating signals. It has since become an integral addition to the statistical pipeline in several recent taxonomic papers (see Grismer et al. 2023, 2024, 2025; Chhin et al. 2024; Quah and Grismer 2025; Quah et al. 2025; and references therein).

A notable advantage of multivariate analyses over univariate analyses is that in some datasets, there may be no statistically significant mean differences among OTUs in character-by-character analyses (e.g., Student t-tests, Welch’s t-test, Mann-Whitney U test, various types of ANOVAs). However, multivariate analyses may recover statistically significant differences among OTU plots because all the character variation in the dataset is analyzed simultaneously, and as such, the data points (i.e., the specimens) will cluster based on their overall similarity to one another. Another notable advantage of multivariate analyses is their ability to detect complex patterns in shape/size change, as well as patterns based on the interaction or combination of traits that univariate analyses cannot recover. These too can have diagnostic value. When morphospatial clustering (plots) aligns with OTUs (or MOTU [molecular operational taxonomic unit] for molecular data) in a phylogeny, it represents a robust inference for testable taxonomic hypotheses.

## ﻿Principal component analysis (PCA)

As noted, PCA is one of the most widely used multivariate analyses in herpetological taxonomy, as well as being one of the oldest methodologies of ordination analyses (Pearson 1901). PCA is an *unsupervised* analysis in that all specimens are treated independently and not grouped *a priori* (i.e., before the analysis is run) according to some criterion such as taxonomy or geography. In the analysis, the raw data for each character from all the OTUs are scaled by dividing the value of each by its standard deviation, thus creating a standard deviation of one for each character. This way the data are analyzed on the basis of correlation not covariance, are weighed equally, and analyzed in proportion to their variance (i.e., a measure of how spread out the data are around the mean). These values are transformed (see below) and plotted onto a multidimensional graph where each character’s data are represented by the character’s own axis or PC. Thus, the number of PCs equals the number of characters in the dataset, and each OTU has a numeric value for each character along each PC. These transformed values are used to construct a Euclidean (dis)similarity matrix comprised of distance values that transform each PC into a linear combination of a new synthetic set of values based on their distances to one another. The synthetic values of the new transformed matrix are then graphically represented in the ordination plot (i.e., the PCA graph). In other words, the Euclidean distance of each individual to all other individuals across all the PCs determines where that individual (now a data point) will be located on the PCA graph. These newly transformed synthetic variables or PC scores—represented as a set of PCs or axes— are arranged such that the first PC (PC1) represents the largest amount (i.e., the spread) of variation in the dataset, PC2 represents the second-most amount of variation, and so forth until all the variation in the dataset has been accounted for.

PCA can use both size-corrected morphometric (see below) and meristic data (i.e., numeric data [numbers]) but not raw untransformed categorical data. However, combining morphometric and meristic *data types* in the same *dataset* should be avoided (see below). There are even arguments for not using discrete data (e.g., meristic [scale counts], transformed categorical data, etc.) in a PCA as opposed to continuous data (e.g., morphometric, physiological, call frequency, etc.) because discrete data do not fulfill the assumptions of linearity (Marhold 2011; Paliy and Shankar 2016). This can present a problem if the range of character variation is large (e.g., a scale count range of 10–100 in a single character); however, expansive character ranges rarely occur in herpetological datasets. Most often—if not always—the degree of variation within the range of an individual character (e.g., a scale count) is so small that the assumptions of linearity are not violated.

PCA is useful in that separate analyses of different data types can be used to infer how each data type explains variation among OTUs independently of other data types. This is important if one is interested in how different OTUs are shaped (i.e., morphometrically related) with respect to one another, what characters are the principal drivers of the different shapes, and how these shapes may or may not correlate with some other aspects of the OTU’s biology. For example, Quah et al. (2025) demonstrated that allopatric populations of the karst-associated species of the Battambang Bent-toed Gecko, *Cyrtodactyluskampingpoiensis*, did not differ significantly in body shape but differed widely in their scale counts suggesting that these different biological systems may be under different selection pressures where a karstic microhabitat may narrowly restrict body shape variation but has no significant bearing on numbers of scales (see Fig, 1B, E and the explanation below). In another example, Grismer and Grismer (2017) demonstrated that in the granite-cave-dwelling Vietnamese Bent-toed Geckos *C.eisenmanae* and *C.grismeri*, the diagnostic characters of larger eyes, longer snouts, longer heads, and longer limbs were correlated along PCs, and were associated with a granite-cave-dwelling lifestyle as opposed to their smaller values in the closely related and sympatric general scansorial species *C.condorensis* and *C.leegrismeri* (Fig. 2).

**Figure 1.**
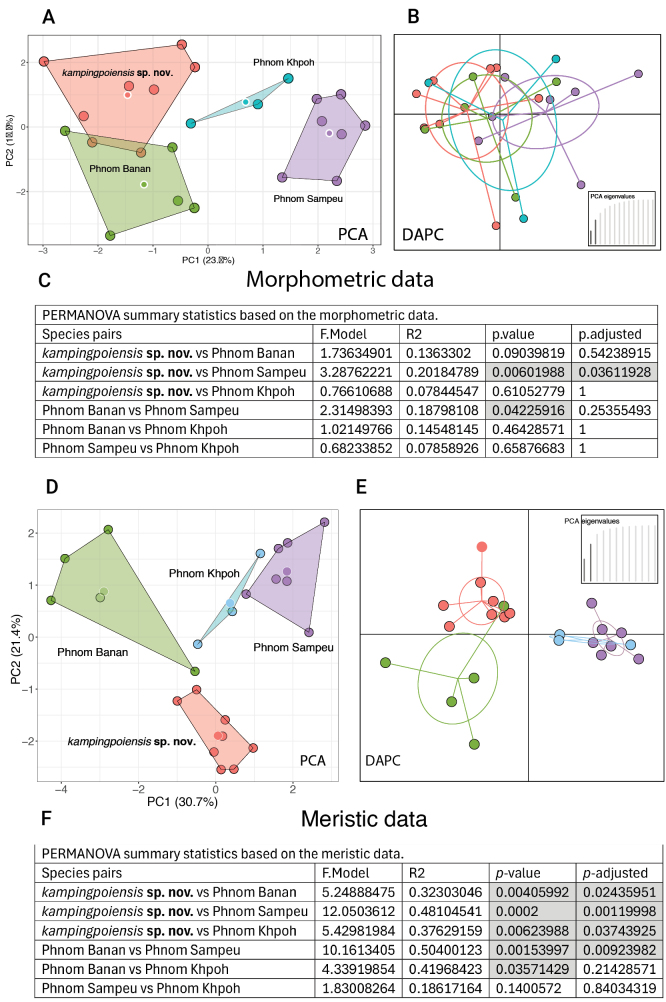
A–C. Morphometric data among allopatric populations of *Cyrtodactyluskampingpoiensis* showing only two pairs of populations bearing significant differences (shaded cells in the PERMANOVA analysis in C) and the wide degree of overlap among all populations in the discriminant analysis of principal components (DAPC) in B. D–F. Meristic data among allopatric populations of *C.kampingpoiensis* showing five pairs of populations bearing significant differences (shaded cells in the PERMANOVA analysis in F. and very little overlap among the populations in the DAPC in E. Circles outlined in white are centroids. Centroids in B and E. are where plot lines converge. Reproduced and modified from Quah et al. (2025).

**Figure 2.**
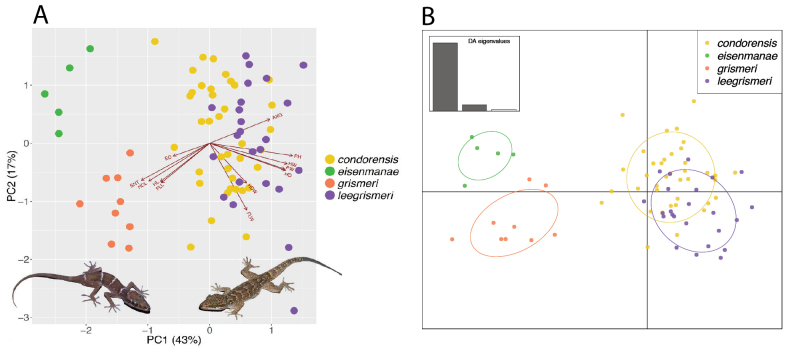
A. PCA biplot illustrating the degree of correlation of large eyes, long snouts, long heads, and long limbs among the granite-cave-dwelling ecomorphs of *Cyrtodactyluseisenmanae* and *C.condorensis* compared to the general scansorial ecomorphs *C.condorensis* and *C.leegrismeri*; B. DAPC plot showing the separation of the granite-cave-dwelling and general scansorial ecomorphs. Illustrations in A are of *C.eisenmanae* (left) and *C.grismeri* (right). AXG = axilla-groin length, PH = pelvic height, HW = head width, PW = pelvic width, HD = head depth, HDW = hindlimb width, FLW = forelimb width, FLL = forelimb length, HL = head length, HDL = hindlimb length, SNT = snout length, and ED = eyeball diameter. Reproduced and modified from Grismer and Grismer (2017).

A discriminant analysis of principal components (DAPC)— part of the *adegenet* R package (Jombart and Collins 2022)—is often performed alongside a PCA. This is another type of ordination analysis that is fundamentally different from PCA and MFA (see below) in that it is a supervised analysis that places individuals into designated groups prior to running the analysis—that is, it *discriminates* the specimens’ group membership beforehand. By doing so, the analysis minimizes within-group variation (smallest variance [i.e., the spread of data points around the centroid]), while maximizing among-group variation (greatest distance among the centroids). DAPC uses the factor loadings of only the most informative PCs, thus reducing the dimensionality of the original dataset even further. It should be noted that the *a priori* groupings required for DAPC do introduce bias, and as such, should be performed as a supporting analysis to PCA instead of using it as the main discriminatory tool.

## ﻿Multiple factor analysis (MFA)

MFA is a global, *unsupervised*, multivariate analysis whose goal is to integrate different kinds of variables/traits/characters (referred to here as data types) describing the same observation. MFA can include categorical (e.g., big or small, blue or red, striped or banded, keeled or smooth, present or absent, etc.) and numeric data (continuous and discrete) in a single dataset (Pagès 2015), thus allowing a nearly total-evidence morphological analysis. Taxonomically, such an analysis is heuristically more informative than PCA in that it simultaneously analyzes the contributions of the different data types to the overall ordination (i.e., plotting) of the OTUs as opposed to a biased analysis using just one data type or another (i.e., “cherry picking” in a sense when more than one data type is available.) In an MFA, each individual/specimen is described by a different set of variables (e.g., 10 dorsal scales, 30 mm hindlimb length; body striped; scales keeled, basal metabolic rate 695) which are structured into separate datasets based on their data type, such as quantitative (i.e., meristic and morphometric—a separate dataset for each numeric data type) and qualitative (categorical—a separate dataset for each categorical data type). The variables in each group are normalized separately (i.e., weighted) and then combined into a single global dataset prior to the final analysis (see below). The normalization step is necessary to balance the influences of each set of variables. In a non-normalized dataset, the structure of the output (i.e., the graph) would be dominated by the variable(s) with the largest amount of variation or, in some cases, statistical variance. In MFA, the contribution of the variables in each dataset is considered when plotting the position of each individual from each OTU.

**Figure 3.**
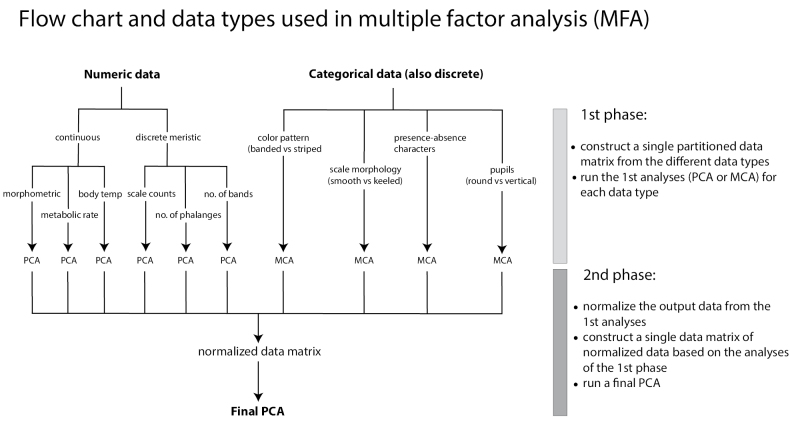
Flow chart of the sequential steps within the 1^st^ and 2^nd^ phases of an MFA and how and where the different numeric and categorical data types are used in the analysis. PCA = principal component analysis and MCA = multiple correspondence analysis.

MFA works in two phases (Table 1, Fig. 3). In the first phase, separate multivariate analyses are carried out for each data type—PCAs for the quantitative data types and multiple correspondence analyses (MCA) for categorical data types. These separate analyses transform the raw data into loading scores along each axis (dimension). In the second phase, the transformed data types are then normalized by dividing the loading scores of each dimension by the square root of the eigenvalue (referred to as the “first singular value”) of the first dimension in the analysis so that the first principal components have the same length. These normalized data types are then combined (i.e., concatenated) to form a global dataset for a final PCA. The normalization process in the second phase ensures that each data type contributes equally to the overall analysis, preventing one data type in the global dataset from dominating due to differences in scale or variance. Therefore, the contribution of each data type is taken into account in the final analysis represented graphically as a scatter plot (Pagès 2015; Kassambara and Mundt 2017).

**Table 1.** Sample MFA dataset comprised of size-corrected morphometric (green), meristic (blue), and three data types of categorical characters (coded yellow, orange, and gray). Each color-coded column of a different data type will be analyzed separately in the first phase of an MFA which will be separate PCAs for green and blue and separate MCAs (for yellow, orange, and gray (see Fig. 3)).

Species	Snout-vent length	Head length	Head width	Head depth	Axilla-groin length	Forelimb length	Hindlimb length	
speciesA	1.897077003	1.049100026	1.13665543	1.514108961	1.323680148	1.139453736	0.905969562	
speciesA	1.851869601	1.085979234	1.160260645	1.548274112	1.31547634	1.153272842	0.963635997	
speciesA	1.853698212	1.057721181	1.152014555	1.52439554	1.320371821	1.135465944	0.931935749	
speciesB	1.900913068	1.05211498	1.152180577	1.522084325	1.326336748	1.152507762	0.971406547	
speciesB	1.891537458	1.075794174	1.131102333	1.511341139	1.322321054	1.153800315	0.895991691	
speciesB	1.851258349	1.040460898	1.151603126	1.52431887	1.311669644	1.166228157	0.902068728	
speciesC	1.72427587	1.058852622	1.135780471	1.514570835	1.325793134	1.15545368	0.93677663	
speciesC	1.897627091	1.062530419	1.130211672	1.518379877	1.325162144	1.174979452	0.961562967	
speciesC	1.851258349	1.032385245	1.172353038	1.508095446	1.325510884	1.148379582	0.933746817	
	Suprlabials	Infralabials	4^th^ toe lamellae	Ventrals	Pattern	Color	Size	Tubercles
speciesA	9	9	32	20	spotted	red	big	absent
speciesA	11	9	37	21	spotted	red	big	absent
speciesA	10	10	34	20	spotted	red	big	absent
speciesB	11	9	34	19	banded	blue	big	present
speciesB	11	9	35	20	banded	blue	big	present
speciesB	10	10	31	20	banded	blue	big	present
speciesC	10	9	30	20	banded	green	small	absent
speciesC	11	10	34	21	banded	green	small	absent
speciesC	10	10	37	20	banded	green	small	absent

There are notable advantages that MFA has over PCA. The first is its ability to integrate different data types into the same analysis. This makes good biological sense because different data types represent different biological systems and, as such, are presumably under different selection pressures (or some may be under no selection pressure at all). Thus, they could be evolving independently but collectively contribute to the overall evolutionary trajectory of the OTU. Running independent analyses on the different data types in the first phase and then normalizing them in the second phase is analogous to using a partition scheme to model the evolution of different genes in a concatenated genetic dataset (or perhaps codons within the same gene), whereas using a single data type in a PCA is analogous to running an unpartitioned analysis. Another advantage is that many species diagnoses omit categorical data because a particular character may not occur in all individuals. For example, in species A, 70% of the individuals may be spotted while 30% are banded and vice versa for species B. This does not prevent MFA from using these characters because *each* individual is coded accordingly (in this case “spotted or banded”). Being that MFA is an unsupervised analysis, each individual is treated independently of every other individual, and individuals will ultimately cluster (plot) accordingly with individuals to which they are most similar. Therefore, in this way, categorical data can be jointly analyzed statistically to inform a multivariate diagnosis. Additionally, categorical data do not have to be binary but can be multistate. For example, species A may have blue spots on the head, the head spots of species B may be green, and those of species C may be red—and these can also occur in varying frequencies. Treating categorical characters in this manner emphasizes their diagnostic utility in a statistically defensible landscape as opposed to their often-anecdotal treatment or even omission.

Another advantage of MFA—as noted above—is that each data type is *normalized* separately and then combined with the other data types so one cannot overleverage the other. When using a mixed dataset of meristic and morphometric characters in a PCA, the different data types are standardized or scaled simultaneously. This treats meristic and morphometric data as a single biological system, which they clearly are not. For example, body parts change allometrically with growth and age, whereas scale counts remain the same throughout life. In fact, Chan and Grismer (2021) provided an empirically tested methodology to reduce the amount of allometric variation in differently-sized individuals of the same OTU. Conflating the biological differences of different data types by running them together in a PCA, will dilute the impact that each data type has on the output of the analysis which will result in the data type with greatest statistical variance overleveraging the others, or in some cases result in overlapping plots bearing no statistically significant differences. The normalization process of the data types in an MFA is not subjected to character bias.

Lastly, MFA automatically handles missing data (i.e., empty cells in the data matrix) when using the mfa() function in FactoMine R (Husson et al. 2017). This function addresses missing data by imputing missing values in both numeric and categorical variables, using methods that consider the structure of the surrounding data and the relationships between variables and groups. For categorical variables, missing data are treated as a new character state. PCA can deal with missing data, but it requires careful consideration and specialized methods to deal with missing values because PCA algorithms are not designed for incomplete data sets and directly applying them to datasets with missing values can lead to inaccurate results. If the PCA input data are not manipulated, then either the specimen(s) or the character(s) bearing the missing value(s) must be deleted from the dataset. This can be especially detrimental if sample sizes are small and further exacerbates all the well-known statistical problems when dealing with small sample sizes. It should be noted that MFA requires at least two data types in the dataset and will not work with a single data type, owing to the analytics of the second phase—in which case a PCA should be used.

**Figure 4.**
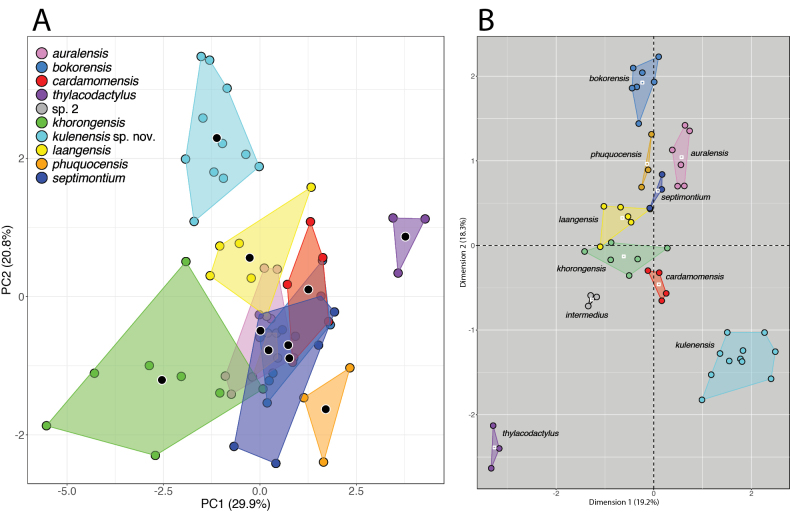
A. PCA of the species of the *Cyrtodactylusintermedius* group based on meristic data. Black circles outlined in white within the plots are centroids; B. MFA of the same species of the *C.intermedius* group based on meristic, size-corrected morphometric, and categorical characters showing greater discrimination among the plots. White squares within the plots are centroids. Reproduced and modified from Grismer et al. (2021).

**Figure 5.**
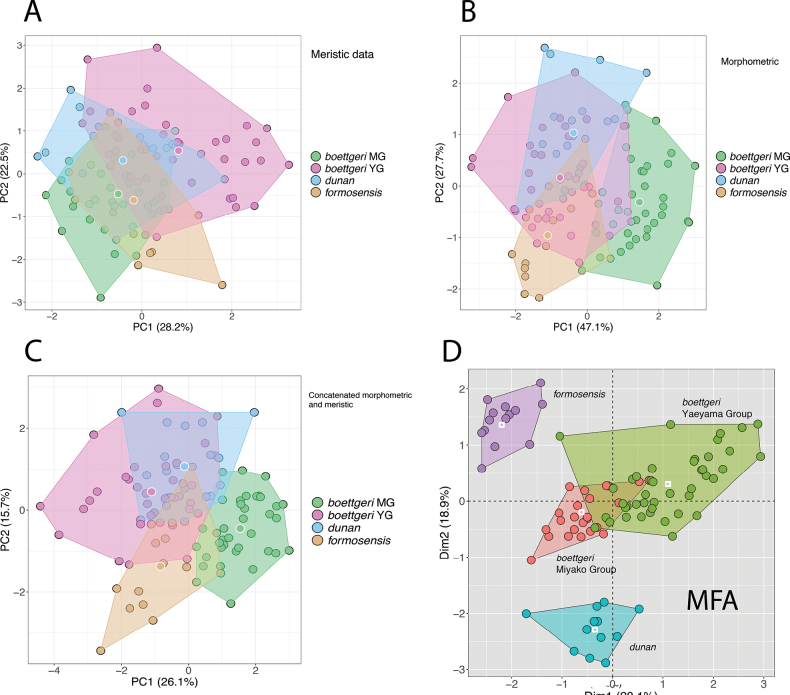
Scatter plots of an east Asian clade of skinks from the genus *Scincella* from the Ryukyu Archipelago, Japan and Taiwan. A. PCA based on meristic data. B. PCA based on size-corrected morphometric data. C. PCA based on a combined meristic and size-corrected morphometric dataset. Circles outlined in white are centroids. D. MFA based on meristic, size-corrected morphometric, and categorical (color pattern) characters. White squares within the plots are centroids. Data for all analyses come from Koizumi et al. (2022), although only graphic D. was published.

In general, MFA has greater discriminating power than PCA largely because it incorporates different data types, including categorical characters, which are usually invariable within OTUs or at least lack the range of variation (after normalization) found in numeric characters and are a valuable source of additional informative character data. For example, Grismer et al. (2021) compared a PCA of meristic data to an MFA using a mixed dataset containing meristic, morphometric, and categorical characters of nine species of Cambodian Bent-toed Geckos of the *Cyrtodactylusintermedius* group, illustrating the greater discriminating power of a nearly total-evidence dataset (Fig. 4). Similar results are obtained when comparing the MFA of an East Asian clade of skinks in the genus *Scincella* to three different PCAs using separate meristic, separate morphometric, and a combined meristic and morphometric dataset (Fig. 5). In the MFA, the categorical data—in this case color pattern—were able to “tease out” morphospatial differences among the OTUs not observable using morphometric and/or meristic data, thus emphasizing the diagnostic utility of statistically analyzed color pattern data. Lastly, another benefit of MFA is that the amount of contributing variation among the different data types, as well as the individual characters, are visualized in bar graphs, providing important information as to which specific characters or data types are the most diagnostically important (Factoextra R; Kassambara and Mundt 2017).

## ﻿Permutation of analysis of variance (PERMANOVA)

**Figure 6.**
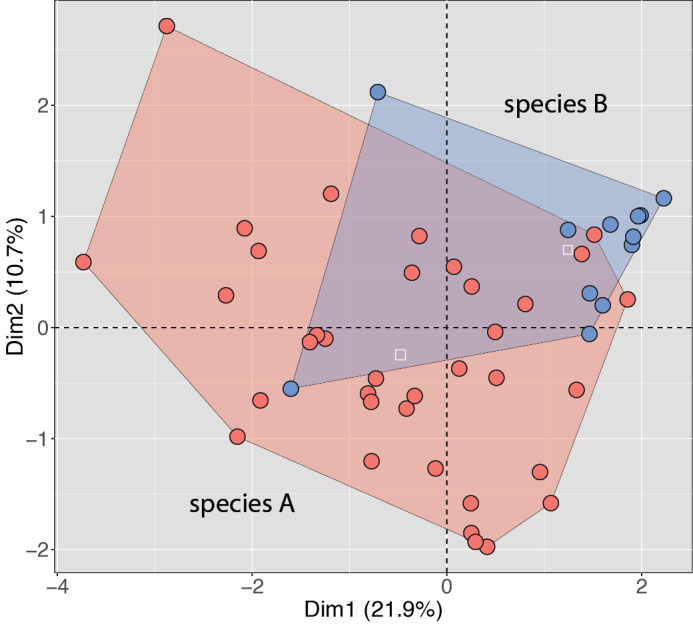
MFA of two unnamed species of *Lycodon* (Quah et al. in prep.) showing wide degrees of plot overlap even though they are statistically significantly different (*p*=2.00E5) based on a PERMANOVA. White squares are centroids.

Objective interpretations of scatterplots are difficult (Peres-Neto et al. 2003) and rarely—if at all—done in herpetological taxonomic papers. Instead, interpretations are usually just “subjective visualization” or simply hand-drawn circles around data points with no consideration of their proximity to each other and with no mention of statistical significance or lack thereof regarding centroid placement or plot overlap. Most often, when OTU plots do not overlap along the ordination of the first two principal components or dimensions, this is considered evidence that the OTUs differ in a taxonomic sense. And the converse is true, if two plots overlap, the OTUs are considered not to differ—not to mention the vague rhetoric describing overlap such as slightly or moderately, etc. However, such claims should not be made in the absence of statistical justification. As with any statistical analysis, significant differences rely heavily on sample size, variance, standard deviation (i.e., the amount of variation of a value around the mean), and standard error (i.e., precision of a sample mean as an estimate of the population mean and used to calculate *p*-values). In the upper PCA plot of Fig. 1A based on morphometric data, the four plots are well-separated from one another although only two population pairs differ significantly from each other (Fig. 1C and see Fig. 1B). In Fig. 1D in the lower PCA of the same populations based on meristic data, the four plots are also well-separated from one another and all but one population pair are significantly different from each other (Fig. 1F and see Fig. 1E). Furthermore, overlapping plots with large sample sizes and small standard deviations may differ significantly (Fig. 6) whereas non-overlapping plots with small sample sizes and large standard deviations may not be significantly different from one another.

One method of objective interpretation is a non-parametric permutation of analysis of variance (PERMANOVA) (Anderson 2001). This is a robust, statistically defensible alternative to subjective interpretations. PERMANOVA from the R packages *vegan* 2.5-3 in R (Oksanen et al. 2020) or *PERMANOAVA* 0.2.0 (Vicente-Gonzalez and Vicente-Villardon 2022) calculates statistical differences among OTUs based on a numeric multivariate dataset (Skalski et al. 2018). The analysis calculates the same Euclidean (dis)similarity matrix used in a PCA or calculates a Euclidean (dis)similarity matrix from MFA dimension loadings, using any chosen number of permutations (I have found 50,000 works well). Although other distance calculations can be employed, I recommend Euclidean distances to be consistent with the PCA or MFA. Based on the (dis)similarity matrices, a pairwise *post hoc* test calculates the differences between all combinations of OTU pairs, generating a Bonferroni-adjusted *p*-value and a pseudo-*F* ratio (*F* statistic). A *p* < 0.05 is considered significant, and a larger *F* statistic (basically representing the ratio of signal to noise) indicates more pronounced group separation. A rejection of the null hypothesis (i.e., that centroid positions are not different from random) signifies a statistically significant difference between OTU pairs.

## ﻿Discussion

In my opinion, a statistically defensible species diagnosis is the *sine qua non* of any diagnosis and this is not simply academic. Such species are paramount for understanding biodiversity, ecological monitoring, informing conservation efforts, effectively and efficiently managing ecosystems, evaluating species-management plans, medical science, and much more (see Hey 2009; Borkent [2021] and references therein). They also preempt taxonomic vandalism by rouge taxonomists (see review in Naish [2025]). Many current diagnoses are little more than differing ranges of meristic characters. Those ranges that do not overlap are usually taken as evidence of species validity. But as noted above, this depends heavily on sample size and variance, which bear on the statistical defensibility and the informativeness of a particular character. Even if character ranges or plots widely overlap but have significantly different mean values or centroid positions, these averages represent statistical models informing us of the different evolutionary tendencies of a character among or between OTUs. Statistically defensible diagnoses are even more important for new species descriptions where genetic data are not available (or if available, frustratingly not used) because without them, the statistically defensible validity of the new species (barring truly cryptic species) remains dubious and its management may be a waste of funds that could go elsewhere.

Multivariate diagnoses are not without some epistemological challenges in that they do not recover characters traditionally used in dichotomous taxonomic keys or that are normally included in a list of diagnostic traits (often true for single characters with overlapping ranges between or among species that have significantly different means.) However, this is a methodological challenge that does not bear on the ontological properties of the species. Among the standard list of written diagnostic characters in the diagnosis section of a species description, it can be simply noted that “*species A* sp. nov. differs statistically (*p* = some value) in morphospace from other species”. The same way that characters bearing significantly different mean values can be listed in the diagnosis section.

**Figure 7.**
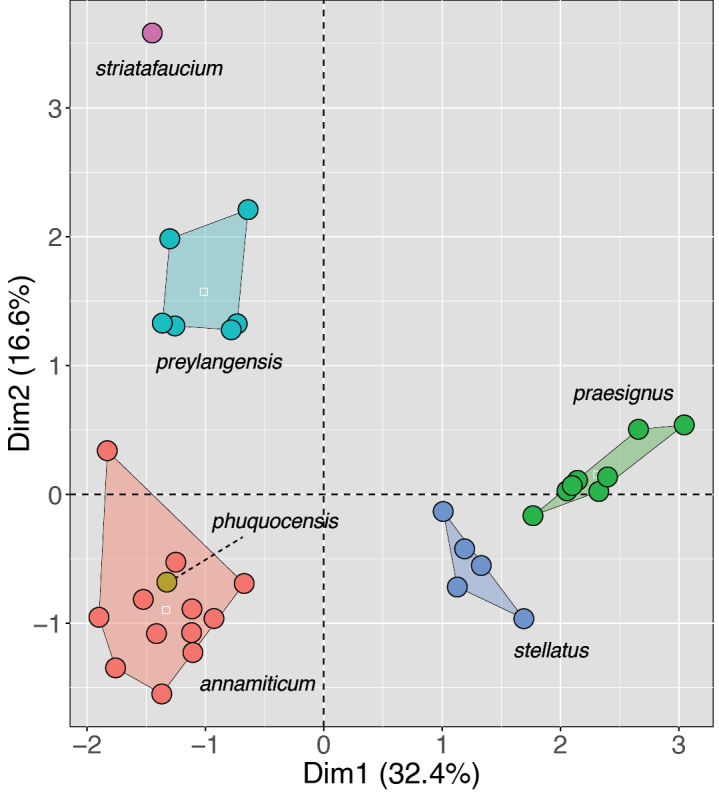
MFA of the *Sphenomorphusstellatus* complex after Quah and Grismer (2025). The placement of the single specimen of *S.striatafaucim* aligns with its phylogenetic separation from all other species in the group. However, this is not the case with *S.phuquocensis*, although it was phylogenetically distinct and well-separated from *S.annamiticum* in a DAPC (Grismer et al. 2019, not shown). White squares are centroids.

Another issue for all statistical analyses is very small sample sizes (*N* < 3)—with *N* = 3, at least a legitimate range, mean, and standard deviation can be calculated. Small sample sizes are not necessarily an insurmountable problem in many cases. In the absence of a recent phylogeny, if the numeric value of an individual character from an underrepresented population falls “well-outside” the range of that of well-sampled putatively close relatives, then that is a solid foundation for building a testable hypothetical diagnosis followed with the acquisition of additional specimens. The same holds for a multivariate approach. If the underrepresented population falls “well-outside” the clusters of well-sampled putative close relatives, then that is an added layer of evidence that aligns with the previously mentioned raw data. If the multivariate analysis is an MFA that includes invariant categorical characters (or only “slightly” variable) in the other sampled taxa, then the diagnostic hypothesis becomes even more robust and is another reason categorical characters should be folded into all statistical operations when possible. Therefore, small sample sizes should not necessarily be dismissed from statistical procedures. In fact, I would opine all the more that if the sample size for one OTU is small, statistical evaluation becomes even more necessary because it can at least inform us where the individuals of the poorly represented OTU plots out with respect to the other closely related species—and this can be the basis for a testable hypothesis (Fig. 7).

In an era of bioinformatics with increasingly complex data-rich and analytically intensive reconstructions of molecular phylogenies and species delimitation methods, it is ironic that the methodology (or lack thereof) for morphologically diagnosing species has not progressed much beyond the 1800s. Chan and Grismer (2021) estimated that from the years 2008–2020, only 34% of the papers dealing with reptile taxonomy included any form of statistical diagnostic analyses, and there were even fewer analyses in amphibian publications (25%). Apparently, this has not improved much (as judged from the herpetological publications in the journal *Zootaxa* just for the year 2024 and see Sharkey et al. 2021 who described 403 new species of wasps with no statistical analyses). It is difficult to say why this is so. Perhaps, it has to do with the fact that taxonomy is no longer considered as important as it used to be and is in a state of crisis (Agnarsson and Kuntner 2007; Sangster and Luksenburg 2015)—endangering the profession itself (Pearson et al. 2011). Alternatively, there may be a societal bias towards the more charismatic taxonomic groups (Troudet et al. 2017), resulting in taxonomists becoming the endangered group (Wägele et al. 2011). More to the point, however, basic taxonomic research is not published in top-tier journals whose publications are necessary to attain academic jobs and promotions. I have been told that some institutions discourage faculty from writing taxonomic papers that are ultimately published in low-impact journals because it lowers the departmental or university average impact factor! Therefore, research emphasis has turned more towards increasingly complex analyses that may not directly contribute to biodiversity conservation—or they often generate exhaustive molecular phylogenies bearing a plethora of unnamed lineages that more often than not, go undescribed. Relegating some of these lineages or populations to ecological ghosts that in some cases have no formal protection does not advance our efforts to conserve biodiversity and leaves them prey to “nomenclatural harvesting” (Denzer and Kaiser 2023). It is hoped that by introducing simple robust statistical procedures here and elsewhere (e.g., Chan and Grismer 2021; and Chan and Grismer in prep.), morphological diagnoses will be brought into the fold of modern science and encourage researchers not to abandon unnamed tips on trees. We cannot protect something if we don’t know it exists.

## ﻿Additional information

### Use of AI

No use of AI was reported.

### Author ORCIDs

L. Lee Grismer https://orcid.org/0000-0001-8422-3698

### Data availability

All of the data that support the findings of this study are available in the main text.
